# Local Anesthetic Use and Intraoperative Pain During Root Canal Treatment: A Cross-Sectional Survey Among Dental Students, Postgraduates, and Endodontists

**DOI:** 10.3390/dj14100647

**Published:** 2026-10-07

**Authors:** Marie Bjørnstad Frøystadvåg, Thea Grambo, Sivakami Rethnam Haug

**Affiliations:** Department of Clinical Dentistry, Section of Endodontics, The Faculty of Medicine, University of Bergen, Årstadveien 19, N5009 Bergen, Norway

**Keywords:** dental education, pulpitis, emergency treatment, previously root-filled tooth, lidocaine, intraoperative pain, local anesthesia, endodontics, endodontic education

## Abstract

**Objectives**: This study investigated the use of local anesthetics (LA) during root canal treatment (RCT) among undergraduate dental students and specialists (postgraduates and specialists in Endodontics). **Methods**: A cross-sectional observation study was conducted among treatment providers, who completed a questionnaire at the end of a treatment visit. **Results**: A total of 134 treatment responses or patient encounters were included. Intraoperative pain (IOP) was reported in 25 (18.7%) responses, whereby 19 (14.2%) received supplementary anesthesia. A statistically significant association was observed between treatment procedure and IOP (*p* < 0.05), with the highest frequency of pain occurring during emergency treatment (55.6%) and the lowest during obturation (10.2%). No statistically significant associations were observed between IOP and pulpal diagnosis, treatment visit, or tooth type. Twenty-nine (21.6%) treatment providers (17.9% dental students and 25.4% specialists) carried out the RCT procedure without the use of LA. The use of LA differed significantly across diagnosis (*p* < 0.001) and treatment procedures (*p* < 0.05). Lidocaine was the most frequently used LA (84.7%). Anesthetic insufficiency (failure or inadequacy) was reported by treatment providers in 21 of 105 treatment encounters (20%) in which LA was administered. IOP was significantly associated with treatment by dental students (OR 7.6, 95% CI: 2.1–27.9; *p* < 0.01) and with the presence of preoperative symptoms (OR 2.9, 95% CI: 1.1–7.9; *p* < 0.05). LA was considered unnecessary by 43.2% of treatment providers in procedures where this response was recorded. **Conclusions**: Operator-reported anesthetic insufficiency was observed in approximately one in five (20%) treatment encounters in which LA was administered. Preoperative symptoms and operator training level may contribute to the occurrence of IOP and should therefore be considered when planning and delivering endodontic treatment.

## 1. Introduction

Teeth are innervated by branches of the fifth cranial nerve, the trigeminal nerve, with the maxillary branch supplying the teeth in the maxilla and the mandibular branch supplying those in the mandible. Two main types of sensory nerve fibers are present in the dental pulp: myelinated A-fibers and unmyelinated C-fibers. Among the A-fibers, A-delta fibers conduct impulses rapidly due to their myelination and transmit pain signals directly to higher centers, resulting in sharp, fast, and well-localized pain. In contrast, C-fibers conduct impulses more slowly and are associated with dull, throbbing, and poorly localized pain, which is more commonly observed in advanced pulpal inflammation extending into the deeper regions of the pulp [1].

An injury to the tooth can induce morphological and structural changes in nerve fibers, including increased density, sprouting, and overexpression of nociceptors [2,3]. Dental caries can significantly increase neuropeptide expression in the dental pulp, contributing to neurogenic inflammation and pain [4]. Furthermore, tissue injury and inflammation may alter both the expression and activity of sodium channels. Sodium channels can be pharmacologically classified based on their sensitivity to the toxin tetrodotoxin. Nociceptor-associated sodium channels are approximately four times less sensitive to lidocaine, and their activity can more than double following exposure to prostaglandin E2 [5]. Increased expression of tetrodotoxin-resistant sodium channels in inflamed pulpal tissue may therefore contribute to LA failure or inadequacy [6]. A “hot tooth” refers to a tooth with symptomatic irreversible pulpitis that is difficult to anesthetize using conventional LA techniques [7]. This condition is associated with pulpal inflammation, increased nociceptor sensitization, and altered sodium channel activity, all of which may contribute to problems associated with LA.

Adequate anesthesia is essential not only for ensuring patient comfort but also for enabling the clinician to work in a controlled and stress-free environment, without concern for unexpected patient movement in response to pain. Furthermore, many patients experience stress and anxiety upon learning that they need root canal treatment (RCT), often due to an expectation of pain. This anxiety can further complicate pain management by lowering the patient’s pain threshold and heightening pain perception [8].

Anesthetic failure or inadequacy refers to insufficient pain control during a procedure. Anesthetic failure is characterized by the absence of the intended anesthetic effect, whereas anesthetic inadequacy refers to a partial or suboptimal block with insufficient depth, quality, or duration, potentially resulting in intraoperative pain (IOP) and the need for supplementary anesthesia [9]. Clinical studies have reported that a single inferior alveolar nerve block achieves a success rate of approximately 75–90% in patients with normal pulps [10]. However, in patients of irreversible pulpitis, failure rates following a single inferior alveolar nerve block have been reported to range from 30% to as high as 80%. In a clinical trial involving 26 patients with irreversible pulpitis in a mandibular molar, all patients (100%) reported lip numbness following an inferior alveolar nerve block; however, 62% still reacted positively to pulp testing, highlighting the discrepancy between subjective soft-tissue anesthesia and pulpal anesthesia [6].

According to the American Association of Endodontists, 15 million teeth receive endodontic treatment each year [11]. Patients undergoing RCT present with a wide range of diagnoses and treatment needs. However, there is limited knowledge regarding LA protocols and clinical practices, as well as the frequency of IOP. The primary aim of this cross-sectional observational study was to compare LA practices during RCT across different operator training levels and stages of treatment. The specific objectives were to investigate the frequency of IOP across different diagnoses and treatment procedures and to evaluate practitioners’ perspectives on the use of LA during RCT.

## 2. Materials and Methods

### 2.1. The Participants

This study was conducted at the Department of Clinical Dentistry, University of Bergen, Norway in accordance with the Strengthening the Reporting of Observational Studies in Epidemiology (STROBE) guidelines for cross-sectional studies. The target population consisted of approximately 90 participants, including fourth- and fifth-year undergraduate dental students, postgraduate candidates in endodontics, and faculty endodontists. A questionnaire was developed using Google Forms (Manufacturer: Google LLC, 1600 Amphitheatre Parkway, Mountain View, CA 94043, USA, launched 2008) and was designed to be completed immediately after the treatment procedure, once the patient had left the clinic. Posters with a QR-code and information about the survey were made available in all treatment cubicles. In addition, a link to the survey was sent via student website directly to the dental students and via email to postgraduate candidates in Endodontics and faculty endodontists. All treatment providers completed the same questionnaire, with outcomes assessed based on the treatment provider’s judgment. Participation was voluntary, and each participant could respond multiple times. By participating in the survey, treatment providers consented to the use of their responses for research purposes. Data collection was conducted anonymously, and no information that could be used to identify or trace the data to individual operators or patients was collected.

### 2.2. Questionnaire

The complete questionnaire is provided as Supplementary Material (Supplementary File). The questionnaire comprised four parts and was kept brief, with a maximum of seventeen questions. These were free text entry, closed-end questions, and open-ended questions. The closed-end questions were with both one-answer options and, when appropriate, several options. In addition, an open-ended response option was provided where applicable, allowing participants to express their views when the predefined response options did not adequately reflect their opinions. An answer had to be registered to questions to proceed to the next question.

Part one:

Part one of the questionnaire was information about the purpose of the study, voluntary participation, anonymous data collection, and contact information for the researchers. Treatment providers were informed that participation in the survey constituted consent to the use of their responses for research purposes. It was also stated that this study was reviewed by the Regional Committee for Medical and Health Research Ethics (REK 616463).

Part two:

The questions in the second part collected information about the practitioner, the patient’s gender, tooth number, symptoms, original diagnosis of the tooth, pulpal and periapical, treatment performed, and use of LA. For pulpal and periapical diagnoses, the World Health Organization (WHO) International Classification of Diseases, 10th Revision (ICD-10) was used. The following ICD-10 codes were included: K04.0, pulpitis (including irreversible, symptomatic, and asymptomatic pulpitis); K04.1, necrosis of pulp; K04.4, acute apical periodontitis (of pulpal origin); K04.5, chronic apical periodontitis; K04.6, periapical abscess with sinus; and K04.7, periapical abscess without sinus and previously root-filled tooth. If the practitioner reported that LA was administered, the questionnaire proceeded to Part 3. If LA was not administered, the treatment provider was directed to Part 4 of the questionnaire. A “yes” response to the question, “Does the patient have symptoms today?” was recorded as the “presence of preoperative symptoms”.

Part three:

This section included questions regarding the type and amount of LA administered, the injection technique used, whether the anesthesia was considered sufficient, and whether supplementary LA was required during treatment. If the practitioner reported that the LA was not sufficient, the response was recorded as LA failure or inadequacy. If the practitioner reported that “supplementary anesthesia had been administered”, this was recorded as “supplementary anesthesia administered”.

Part four:

The final section focused on patients’ symptoms and treatment providers’ perspectives on the use of LA. Participants were asked, “Did the patient experience pain during treatment?” A “yes” response was recorded as the “presence of IOP”. They were also asked, “Do you think LA was necessary during the treatment?”, with the response options “yes, no, perhaps yes, perhaps no, and others”. An optional free-text field was provided at the end of the questionnaire for participants to add comments or additional information.

The questionnaire was pilot tested by at least five specialists and five dental students to evaluate for clarity and feasibility. The test survey was tested electronically as the main survey. This process ensured that the electronic survey functioned as intended, that the response options were clearly formulated, that the survey could be completed within a reasonable amount of time, and that all questions were easily understood. The feedback obtained regarding question clarity, interpretation, and usability was used to make minor modifications. The pilot testing confirmed that the questionnaire was clear and feasible to implement.

### 2.3. Collection of Data

A QR code providing information about this study was placed in each treatment cubicle. Data were collected from September 2024 through December 2025. During the data collection period, no changes were made to the clinical protocols, level of supervision, or availability of anesthetic agents. Although the student cohorts changed over time, this provided the additional benefit of increasing the response to the questionnaire.

### 2.4. Statistics

Data were exported from Google Forms into the IBM Statistical Package for Social Sciences (SPSS, IBM Corp. Released 2017. IBM SPSS Statistics for Windows, version 25.0, Armonk, NY, USA: IBM Corp.). Before analyzing, the data were coded, controlled, and checked against the responses to ensure accuracy. Because treatment provider identifiers were not collected due to the anonymous nature of the data collection, clustered analyses, such as mixed-effects regression models or generalized estimating equations, could not be performed. Data for the dependent variable treatment providers were dichotomized to students (fourth- and fifth-year dental students) and specialists (postgraduate candidates in Endodontics and faculty endodontists) for Pearson’s chi-square test. A Fisher–Freeman–Halton exact test was performed for “IOP” and “use of LA” since several categories contained very small numbers. Univariable analyses were performed to evaluate factors associated with IOP during treatment encounters, with results presented as unadjusted odds ratios and 95% confidence intervals. The level of significance was set at 5%. Fourteen treatment providers provided free-text comments. Given the small number of responses, the comments were systematically categorized and are presented descriptively in Section 3.

### 2.5. Ethics

The research protocol was approved by the Department of Clinical Dentistry. The requirement for informed consent was waived by the Regional Committees for Medical and Health Research Ethics West, Norway (REK 616463), as no directly identifiable patient information was collected and the information recorded in the questionnaire could not be used to trace or identify individual patients. Throughout this process, all regulations for GDPR, as legally mandated by the EU for all EU and EEA countries, were strictly adhered to.

## 3. Results

### 3.1. Survey Responses

A total of 137 responses were registered. Three treatment-visit responses were excluded from the analysis because they contained inadequate information or involved treatment procedures that were outside the scope of this study.

**Figure 1:**
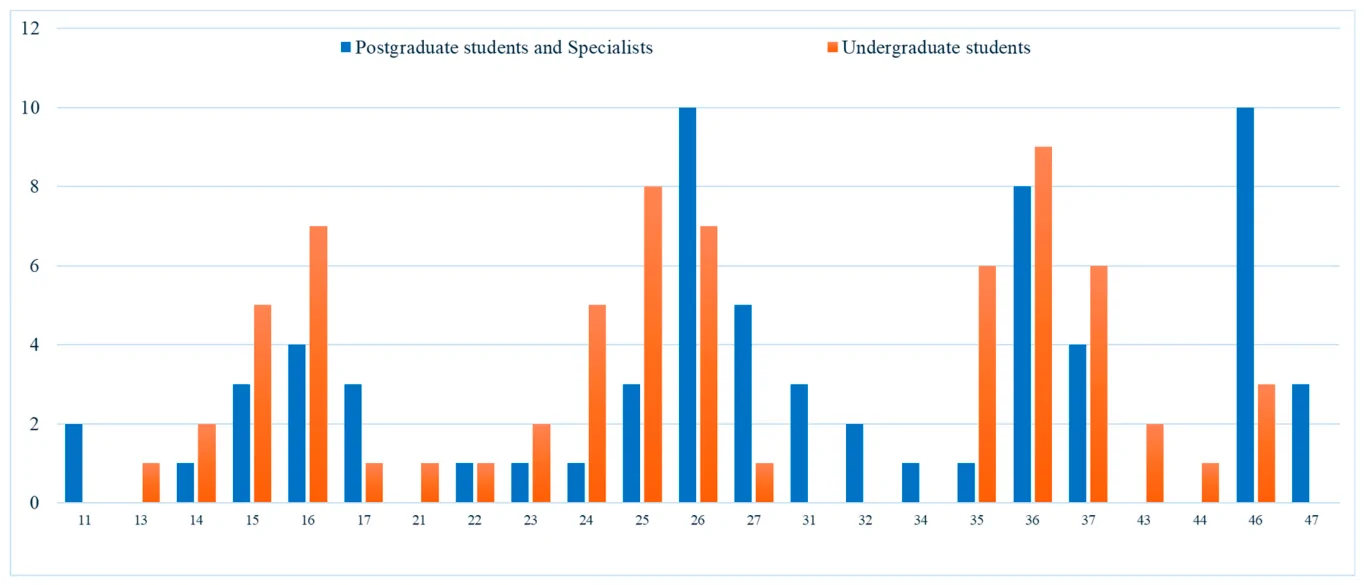
Number of teeth (y-axis) treated by dental students and specialists according to FDI tooth numbering system (x-axis).

Demographic data and patient characteristics for the 134 treatment-visit responses included in this study are presented in Table 1. Diagnosis was registered during the examination and diagnosis visit, which may not be the treatment visit when the response was registered. Frequency of teeth treated by the two cohorts is presented in Figure 1.

### 3.2. Intraoperative Pain

Intraoperative pain was reported in 25 (18.7%) of 134 treatment encounters, of which 19 (14.2%) received supplementary anesthesia (Table 2). The highest frequency of IOP was observed in mandibular molars (10/44, 22.7%), followed by mandibular premolars and maxillary anterior teeth (both 22.2%). The results indicate that the proportion of patients experiencing pain varied across the different treatment procedures, with emergency treatment showing the highest proportion of pain (55.6%) and obturation the lowest proportion (10.2%). There was a statistically significant association between treatment procedure and the occurrence of IOP, with emergency procedures showing a significantly higher occurrence of IOP compared with other treatment procedures (*p* < 0.05). Patients with a diagnosis of pulpitis experienced IOP more frequently (30%) than those presenting with pulpal necrosis (12.5%) and a previously root-filled tooth (18.2%). There were no significant differences between diagnosis, treatment visit, or tooth types.

### 3.3. Use of Local Anesthetics

Twenty-nine (21.6%) treatment providers (17.9% undergraduate students and 25.4% of specialists) carried out procedures without any LA. The use of LA differed significantly according to pulpal diagnosis (*p* < 0.001). LA was administered in 92.5% of teeth with pulpitis, 81.9% of teeth with pulpal necrosis, and 36.4% of previously root-filled teeth (Table 3). The use of LA differed significantly across treatment procedures (*p* < 0.05). LA was administered in all emergency treatments (100%), compared with 78.4% of cavity preparations, 89.7% of instrumentation procedures, and 65.3% of obturation procedures. There were no significant differences between treatment providers, treatment visits, tooth location, or between maxilla and mandible on use of anesthesia. IOP was recorded on four treatment visits where LA was not administered. All four had a diagnosis of a previously root-filled tooth, three were undergoing obturation, and one instrumentation. None of these four received LA during treatment.

The most frequently used LA during treatment visits was lidocaine (20 mg/mL lidocaine hydrochloride with 12.5 µg/mL adrenaline, Xylocaine, Dentsply DeTrey GmbH, Konstanz, Germany) (Table 4). Lidocaine was administered as an inferior alveolar nerve block on mandibular teeth. The second most common LA was articaine (40 mg/mL articaine with 5 µg/mL adrenaline, Septocaine, Septodont, Saint-Maur-des-Fossés, France) followed by mepivacaine (30 mg/mL mepivacaine hydrochloride, Scandonest 3% Plain, Septodont, Saint-Maur-des-Fossés, France) and prilocaine (prilocaine hydrochloride 30 mg/mL, felypressin 0.54 µg/mL, Citanest Dental Octapressin, Dentsply, Dentsply DeTrey GmbH, Konstanz, Germany). Articaine was administered only as an infiltration anesthesia.

### 3.4. Anesthetic Insufficiency (Failure or Inadequacy)

**Figure 2:**
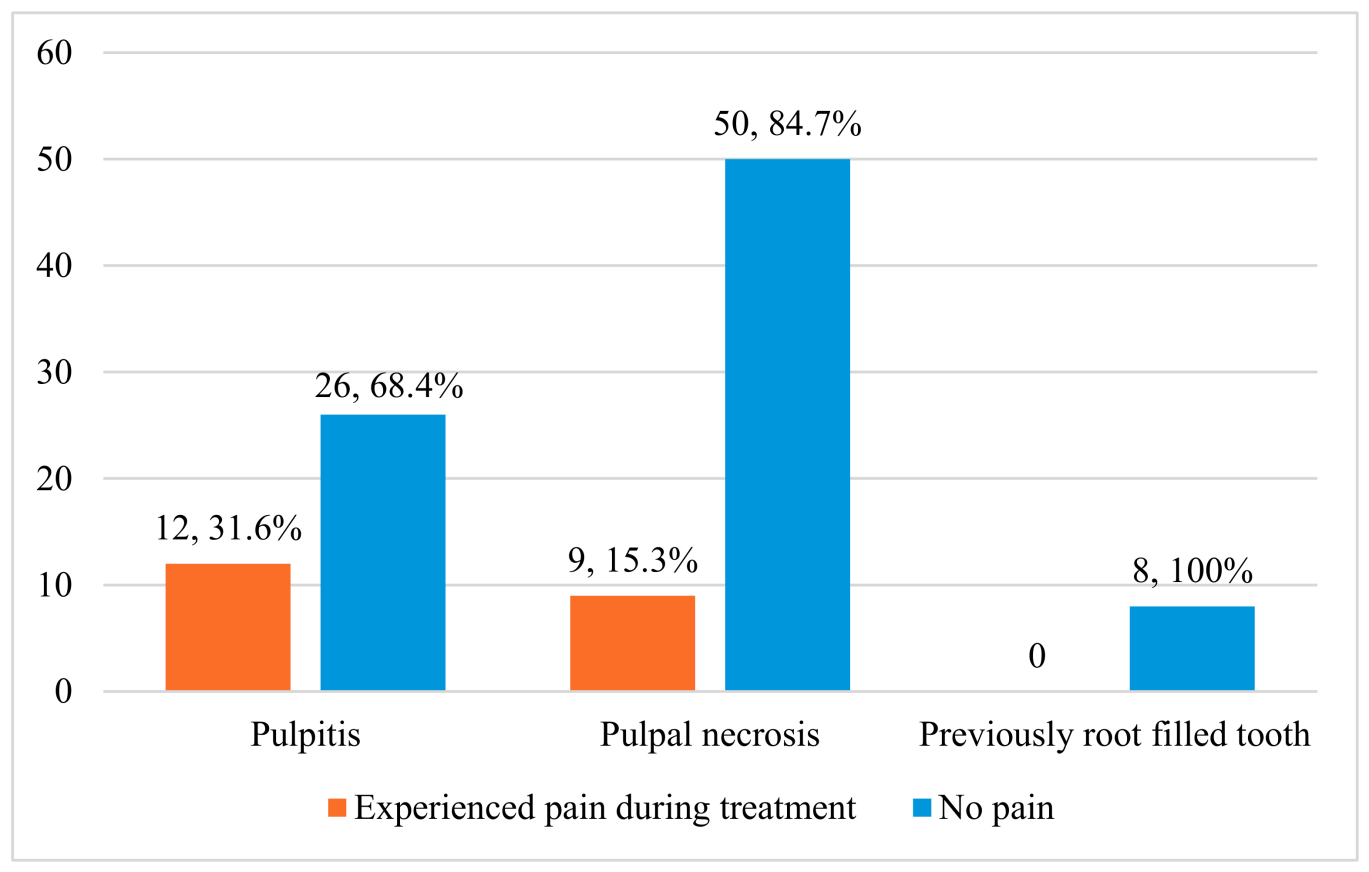
Number and percentage of treatment encounters (n = 105) where IOP was experienced after receiving LA categorized according to diagnosis.

Anesthetic insufficiency (failure or inadequacy) was reported by the operators in 21 (20%) of the 105 patients who received LA. Of these 21 patients, 57.1% had pulpitis as a diagnosis and 42.9% had necrotic pulp (Figure 2). A total of 19 out of 105 patients (18.1%) received supplementary anesthesia. Eight patients received intrapulpal anesthesia on the following teeth: 5 mandibular molars, 1 mandibular premolar, and 2 maxillary molars. Patients experienced IOP during all types of treatment procedures. Univariable analysis showed that IOP was experienced by patients treated by dental students, odds ratio of 7.6 (*p* < 0.01), and with preoperative symptoms, odds ratio of approximately 3 (*p* < 0.05) (Table 5).

### 3.5. Gender Differences

There were no gender differences in pain experience. However, significantly more female patients received LA (*p* = 0.01).

### 3.6. Self-Reflection

When asked if there was a need for anesthesia during this specific treatment procedure, 42 (31.3%) treatment providers answered “no” and 16 (11.9%) answered “perhaps no”.

### 3.7. Comments

Fourteen treatment providers commented to further clarify patients’ pain experiences. Two participants reported difficulty achieving anesthesia, with the LA described as not working as expected; these treatment encounters were recorded as LA failures. Both cases involved mandibular molars diagnosed with pulpitis. One occurred during emergency treatment, while the other occurred during instrumentation visit. Three participants reported having to administer supplementary anesthesia, and that it was effective. Four participants described mild pain or transient sensations experienced by the patient, including discomfort during rubber dam application, a mild transient electric shock-like sensation in the distobuccal canal, transient sensation in the distal canal, and pain during instrumentation in the apical 2 mm of a canal. Five participants provided comments describing aspects of the treatment procedure unrelated to pain or LA.

## 4. Discussion

The main findings of this study are that despite RCT generally being a painless procedure, one-fifth of the patients experienced IOP even when LA was administered. Significantly more emergency treatment and treatment visits to dental students resulted in IOP. Interestingly, LA was not administered for more than one-fifth (21.6%) of treatment procedures, and 43.2% of treatment providers self-reflected that there was “no need” or “perhaps no need” for LA for the specific procedure when the treatment response was registered. LA was used substantially less frequently on previously root-filled teeth and during obturation. The most frequently used LA is lidocaine, followed by articaine.

The normal and inflamed dental pulp is a densely innervated organ [1]. In contrast, a tooth with a necrotic pulp or a previously root-filled tooth lacks both vital pulpal tissue and functional nerve fibers. Consequently, nociceptive signals can no longer be generated or transmitted through the trigeminal sensory pathways to the thalamus and cerebral cortex. Furthermore, periapical lesions are not as densely innervated as a normal or widened periodontal ligament as in acute apical periodontitis [12]. This theory is further corroborated by a study in which RCT was performed without LA in 80 canals (40 necrotic and 40 previously root-filled teeth). No patients experienced pain during working length determination or instrumentation, leading the authors to conclude that LA may not be necessary for teeth with necrotic pulp or during retreatment [13].

In this study, IOP was experienced during various treatment procedures by 18.7% of patients receiving RCT. While these figures are not directly comparable due to the study design, it is less than another study which reported more than double the number of patients, 46%, experiencing mild, moderate, and severe pain during RCT with a mean pain level of 1.2 ± 0.8 using a visual analogue scale (VAS) from 0–10 [8]. The treatment procedures in this study were broadly categorized into four groups: emergency treatment, access cavity preparation, instrumentation, and obturation. Retreatment procedures were included in these treatment categories. Emergency treatment consisted of pulpotomy or root canal debridement, as established from a previous study [14]. More than half the treatment visits (55.6%) related to emergency treatment experienced IOP, which is significantly higher when compared with other treatment procedures such as 16.2% during access cavity preparation, 23.1% during instrumentation, and 10.2% during obturation. Similarly, IOP was more frequent during the first appointment (20.4%) compared to third appointment (5.3%). No patients who received LA experienced pain during treatment on a previously root-filled tooth.

**Figure 3:**
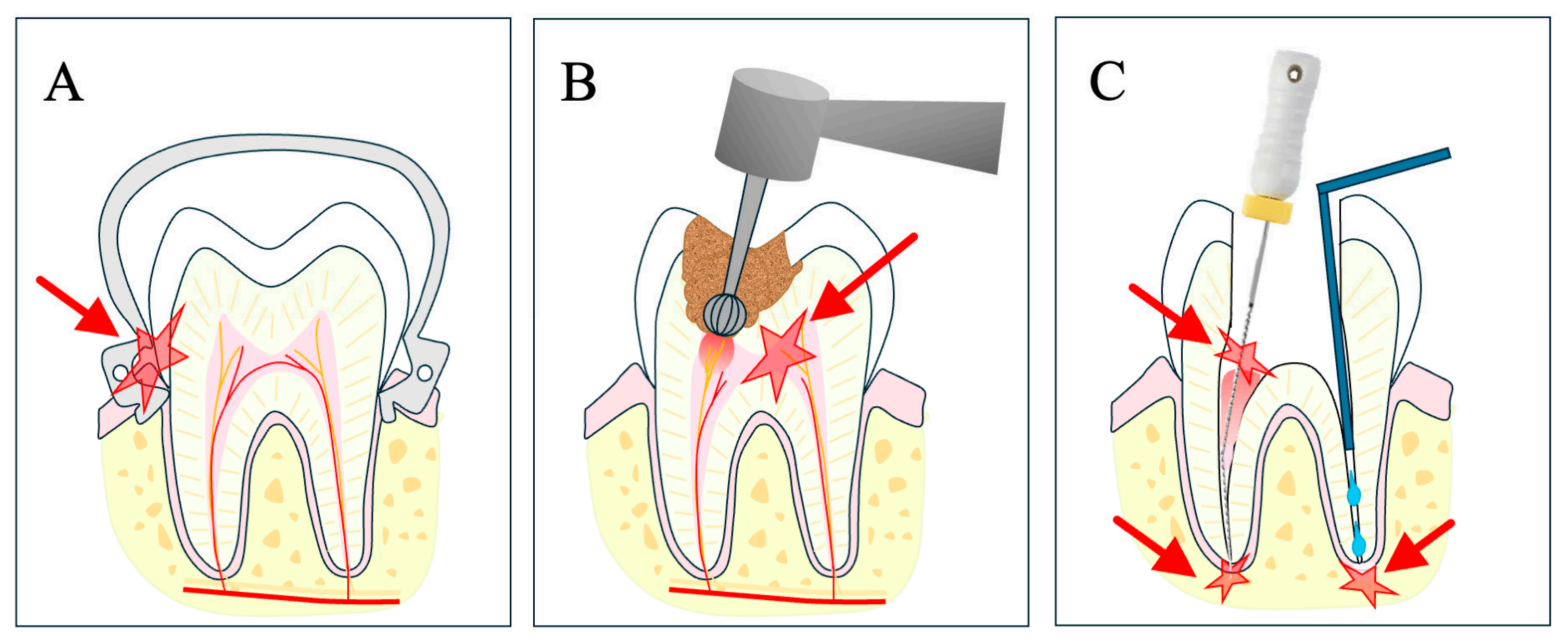
(**A**–**C**) Schematic illustration of common anatomical regions and clinical procedures that may contribute to pain sensation during root canal treatment. (**A**) Pain sensation from periodontium during placement of the rubber dam clamp (red arrow, star), (**B**) during access cavity preparation, patients can experience pain despite the administration of LA, especially in teeth with symptomatic irreversible pulpitis (red arrow, star), (**C**) a missed canal in a previously root-filled tooth may contain vital pulp tissue capable of causing pain. During chemomechanical preparation, overinstrumentation and extrusion of dentinal debris or irrigants beyond the apex may stimulate the innervated periodontal ligament and cause pain. Extrusion of calcium hydroxide can cause severe pain and tissue or nerve damage, while extruded sealer may activate periapical nociceptors through direct stimulation or the release of inflammatory mediators (red arrows, stars).

Pain is a subjective experience and is therefore influenced by many factors, including ethnicity, personality, and physical and psychological factors [15,16]. In this study, IOP was experienced by four patients with previously root-filled teeth. This is because pain sensation during RCT can originate from different regions (Figure 3). Regardless of the pulpal status, the stainless-steel rubber dam clamp used to stabilize the rubber dam may pinch the cervical gingiva and cause pain. Studies show that the placement of rubber dam clamps may be associated with pain and discomfort, especially in children. Therefore, LA may be used to minimize discomfort associated with rubber dam clamp placement [17] (Figure 3A).

During access cavity preparation, patients can experience pain despite the administration of LA, especially in teeth with symptomatic irreversible pulpitis [18] (Figure 3B). Furthermore, a previously root-filled tooth may have vital pulp tissue in a missed canal causing pain during canal negotiation and instrumentation (Figure 3C). This may explain why one of the four patients who did not receive LA on a previously root-filled tooth experienced pain. During chemomechanical preparation, overinstrumentation and extrusion of intracanal dentine debris and irrigants into the periradicular tissue may occur and cause pain sensation since the apical periodontal ligament is innervated [19]. Extrusion of calcium hydroxide, a common intracanal medicament, can cause excruciating pain and tissue and nerve damage when extruded beyond the apical foramen [20]. Ideally, the obturation material should reach the apex of the tooth without extruding into the periradicular tissue. When sealer is extruded into the periradicular area, either direct stimulation or release of inflammatory mediators can activate nociceptors in the periapical region, potentially causing pain (Figure 3C).

In this study, patients who attended the appointment with symptoms had 3 times (odds ratio 2.91 and 95% CI 1.1–7.9) more chance of experiencing IOP than patients without preoperative symptoms. This is in agreement with Aggarwal et al., who showed a positive correlation between the values of preoperative symptoms and IOP [21]. Patients reporting high preoperative pain scores on a VAS may have a greater likelihood of LA failure or inadequacy [21]. In another study, success with inferior alveolar nerve block was achieved in only 48% of patients with moderate-to severe preoperative pain on a mandibular molar [22]. Similarly, Khan et al. reported that one-third (33.3%) of patients with symptomatic irreversible pulpitis and acute apical periodontitis required supplementary anesthesia, administered via intraligamentary or intraosseous injection, despite receiving 3.6 mL of various anesthetic combinations. Consistent with the findings of Khan et al., we did not observe any significant gender differences in pain experience in the present study [23]. However, in our study, IOP was observed more frequently in mandibular molars., notably in both encounters in which the provider described the anesthetic as not working as expected.

Resistance to LA in certain medical situations may be related to several factors, including genetic predisposition, preconditioning, and opioid consumption [24,25]. To our knowledge, none of the patients in this study belonged to these categories. As part of the standard of care, all patients were assessed for adequate anesthesia before the commencement of treatment.

One major concern highlighted in this study is the substantially higher frequency of reported incidence of IOP by undergraduate students. The incidence was approximately seven times greater compared with postgraduate candidates and specialists (odds ratio 7.62, 95% CI 2.1–27.9, *p* = 0.002). The wide confidence intervals indicate limited precision in the estimated odds ratios, likely reflecting the relatively small sample size. Therefore, the magnitude of the observed associations should be interpreted with caution. Potential confounding factors that may have contributed to these findings include diagnosis, treatment procedure, tooth type, injection technique, case complexity, and anesthetic dose. Therefore, the observed association may not be attributable solely to the operator’s level of training. Another contributing factor may be the duration of action of the LA, which could have influenced the occurrence of IOP. Dental students often treat patients during a session that lasts for 150 min. Although treatment duration was not assessed in this study, procedures performed by students typically require more time. In addition to their limited clinical experience, each treatment step must be approved before proceeding to the subsequent stage, potentially further prolonging the overall treatment process. Typically, lidocaine has a duration of action of approximately 55–90 min, whereas articaine generally provides anesthesia for 60–120 min [26]. Given these durations, it is speculated that patients may not have adequate anesthesia towards the end of a treatment session during prolonged duration. This is in agreement with another study where significantly more patients reported absence of pain when the treatment time was within 45 min [8]. Another reason could be that dental students may not have the optimal injection technique when administrating LA nor give adequate amount.

LA was administered in a total of 105 treatment visits. The most frequently used LA was lidocaine, followed by articaine, mepivacaine, and prilocaine. The most common delivery method was infiltration (67.4%), followed by inferior alveolar nerve block (24.8%), intrapulpal (6.2%), and intraligamentary (1.6%). All mandibular teeth received an inferior alveolar nerve block with lidocaine. In certain situations, infiltration with articaine was placed in addition to an inferior alveolar nerve block. Articaine is the newest addition to the class of LA and was approved by the Food and Drug Administration in April 2000 [27]. A potential misconception is that articaine 4% can more likely lead to paresthesia due to nerve blocks compared to lidocaine 2% [28]. According to Pogrel [28], nerve blocks can cause permanent damage to the nerves from 1 in 20,000 to 850,000 patients, independent of the LA used. Permanent nerve damage, which included paresthesia and dysesthesias, was associated with 35% of lidocaine and 29.8% of articaine usage [28]. However, the study also implicated that use of articaine for inferior alveolar nerve block is generally limited.

Another concern in this study is the higher number of intrapulpal compared with intraligamentary. Intrapulpal is considered to be the most painful injection, which has a relatively short duration of effect, approximately 15–20 min. Even though intrapulpal is considered the last option in achieving adequate anesthesia, the result in this study shows a higher percentage in use of intrapulpal (6.2%) compared to intraligamentary injection (1.6%). Intraligamentary injection has a success rate as high as 98.4% [29]. This is also a good supplementary option when there is a problem with achieving full anesthesia of a tooth following intraosseous and the use of computer-controlled anesthesia delivery systems, such as the Wand. However, it has been claimed that supplementary injections, such as intraligamentary and intraosseous, do not provide adequate anesthesia in approximately 5–10% of mandibular teeth diagnosed with symptomatic irreversible pulpitis. In this study, most of the teeth receiving intrapulpal were on mandibular teeth and molars. A recent randomized controlled clinical trial using only articaine as LA reported that 100% successful pulpal anesthesia was not achieved in patients with symptomatic irreversible pulpitis undergoing treatment of maxillary molars. The study further demonstrated that increasing the volume of LA and supplementing buccal infiltration with a palatal infiltration, although associated with increased injection-related discomfort to the patient, improved the success rate of pulpal anesthesia [30]. These findings suggest that the ability to recognize the need for additional anesthetic techniques and to administer them appropriately may develop with increasing operator training and clinical experience, particularly among dental students.

Although LA is generally reliable, its effectiveness has not always met expectations in certain clinical situations. Reducing inflammation by premedicating patients with at least 400 mg of ibuprofen or corticosteroids before delivery of LA has been shown to positively affect the success rate of LA [31,32,33]. Adequate knowledge and patient education, particularly when treating mandibular molars with preoperative symptoms, can help prepare both clinicians and patients, leading to a more predictable and less stressful experience. Scheduling the treatment within the effective duration of the LA further ensures the likelihood of a painless procedure. The finding that approximately one in five treatment providers—including undergraduate students, postgraduate candidates, and specialists—did not administer LA, together with the observation that two in five providers considered LA unnecessary during the treatment procedure, further supports the notion that RCT is, for the most part, a relatively painless procedure. However, the findings from this study should not be misinterpreted as advocating for the non-use of LA during RCT. Apart from eliminating the experience of IOP, LA also alleviates gag reflex when taking radiographs, promotes patient confidence, and reduces treatment-related anxiety.

The relatively low frequency of IOP reported in this study may be explained by the study design, as we did not specifically include patients presenting with preoperative symptoms; rather, we included all patients undergoing RCT at different stages of treatment. Thus, our findings add to the existing literature by providing a more representative overall impression of the likelihood of experiencing pain during an RCT appointment, as well as potential factors associated with IOP. This also highlights the clinical question of whether LA is necessary for all RCT procedures or whether its use should be tailored according to the clinical situation and patient preference.

There are several limitations associated with this single-center study. First, because data were collected anonymously, it was not possible to determine how many treatment encounters were contributed by individual treatment providers or whether the same patient contributed to the dataset on multiple occasions. Second, the questionnaire relied on clinician-judged assessments and not responses directly from the patient. If a patient experienced pain but did not report it to the treatment provider, the occurrence of IOP would not have been recorded. In addition, a supervised student may report IOP differently than a specialist. Furthermore, pain was recorded as an operator-reported binary assessment of observed or communicated pain, not a direct patient-reported measure of pain intensity. The lack of statistical significance should not necessarily be interpreted as evidence that no differences exist, as the relatively small number of observations, particularly the limited number of pain events, may have reduced the statistical power to detect meaningful differences. Because reporting was voluntary, difficult treatment encounters may have been underreported, introducing a potential reporting bias. The diagnosis was established at the first visit; therefore, its relevance and potential impact on intraoperative pain during subsequent treatment stages may be limited. The questionnaire also did not require treatment providers to specify when during the treatment procedure IOP occurred, although some participants provided this information in the free-text comments. As providing comments was optional, this information was not available for all cases. Furthermore, the distribution of treatment providers was not balanced, as the dental school had a larger number of fourth- and fifth-year dental students than postgraduate candidates and faculty. Consequently, potential clustering weighted more heavily on one side of the dataset. Because every encounter is treated as independent, the confidence intervals and *p*-values are narrower than they should be. Finally, as the focus of this study is dental education, treatment providers were categorized as dental students or postgraduate candidates and specialists according to their level of training. Postgraduate candidates may not necessarily possess the same level of clinical competence as specialist endodontists.

Future research should aim to optimize clinical protocols and support the development of novel anesthetic agents that are predictable and effective, especially in patients with preoperative symptoms.

In conclusion, operator-reported anesthetic insufficiency (failure or inadequacy) was observed in approximately one in five (20%) treatment encounters in which LA was administered. Preoperative symptoms and operator training level may contribute to the occurrence of IOP and should therefore be considered when planning and delivering endodontic treatment.

## Figures and Tables

**Table 1:** Demographic data and patient characteristics (n = 134).

Categories	Variables	Number
		(Percentage)
**Treatment**	Dental students (4th year, n = 61; final year, n = 6)	67 (50)
**provider**	Specialists (postgraduates, n = 16; endodontists, n = 51)	67 (50)
**Patients’**	Male	64 (47.8)
**gender**	Female	70 (52.2)
**Pulpal**	Pulpitis (irreversible, symptomatic and asymptomatic)	40 (29.9)
**diagnosis**	Pulpal necrosis	72 (53.7)
	Previously root-filled tooth	22 (16.4)
**Periapical**	No periapical lesion	49 (36.6)
**diagnosis**	Acute apical periodontitis	11 (8.2)
	Chronic apical periodontitis	50 (37.3)
	Periapical abscess with sinus tract	22 (16.4)
	Periapical abscess without sinus tract	2 (1.5)
**Symptoms**	Presence of preoperative symptoms	30 (22.4)
**Dental arch**	Maxillary	74 (55.2)
	Mandibular	60 (44.8)
**Tooth type**	Anterior	16 (11.9)
	Premolar	36 (26.9)
	Molar	82 (61.2)
**Type of**	Emergency treatment	9 (6.7)
**treatment**	Access cavity preparation	37 (27.6)
	Instrumentation	39 (29.1)
	Obturation	49 (36.6)
**Treatment**	First	54 (40.3)
**visits**	Second	47 (35.1)
	Third	19 (14.2)
	More than third	14 (10.4)

**Table 2:** Number and percentage of treatment visits (n = 134) with intraoperative pain (IOP) categorized by diagnosis, treatment procedure, the specific treatment visit, and tooth types (* *p* < 0.05).

Categories	Variables	Experienced IOP During Treatment, n (%)		
		Yes	No	Total
Pulpal	Pulpitis	12 (30)	28 (70)	40
diagnosis	Pulpal necrosis	9 (12.5)	63 (87.5)	72
	Previously root-filled tooth	4 (18.2)	18 (81.8)	22
Periapical	No periapical lesion	10 (20.4)	39 (79.6)	49
diagnosis	Acute apical periodontitis	4 (36.4)	7 (63.6)	11
	Chronic apical periodontitis	5 (10)	45 (90)	50
	Periapical abscess	0 (0)	2 (100)	2
	Periapical abscess with sinus tract	6 (27.3)	16 (72.7)	22
Treatment	Emergency treatment *	5 (55.6)	4 (44.4)	9
procedure	Access cavity preparation	6 (16.2)	31 (83.8)	37
	Instrumentation	9 (23.1)	30 (76.9)	39
	Obturation	5 (10.2)	44 (89.8)	49
Treatment	First	11 (20.4)	43 (79.6)	54
visit	Second	9 (19.1)	38 (80.9)	47
	Third	1 (5.3)	18 (94.7)	19
	More than third	4 (28.6)	10 (71.4)	14
Tooth	Maxillary anterior	2 (22.2)	7 (77.8)	9
type	Maxillary premolar	3 (11.1)	24 (88.9)	27
	Maxillary molar	7 (18.4)	31 (81.6)	38
	Mandibular anterior	1 (14.3)	6 (85.7)	7
	Mandibular premolar	2 (22.2)	7 (77.8)	9
	Mandibular molar	10 (22.7)	34 (77.3)	44

**Table 3:** Number and percentage of treatment visits (n = 134) where local anesthesia (LA) was administered, categorized according to pulpal diagnosis, treatment procedure, and specific treatment visit (** *p* < 0.001, * *p* < 0.05).

Categories	Variables	Use of LA, n (%)		
		Yes	No	Total
Pulpal	Pulpitis	38 (95.0)	2 (5.0)	40
diagnosis	Pulpal necrosis	59 (81.9)	13 (18.1)	72
	Previously root-filled tooth **	8 (36.4)	14 (63.6)	22
Treatment	Emergency treatment *	9 (100)	0 (0)	9
procedure	Access cavity preparation	29 (78.4)	8 (21.6)	37
	Instrumentation	35 (89.7)	4 (10.3)	39
	Obturation	32 (65.3)	17 (34.7)	49
Treatment	First	45 (83.3)	9 (16.7)	54
visit	Second	35 (74.5)	12 (25.5)	47
	Third	15 (78.9)	4 (21.1)	19
	More than third	10 (71.4)	4 (28.6)	14

**Table 4:** Anesthetic usage during treatment visits (n = 105). * Several teeth received more than one mode of application. ** Each cartridge contains 1.8 mL of anesthetic solution.

Categories	Variables	Number
		(Percentage)
**Type of**	2% Lidocaine	89 (84.7)
**anesthesia**	4% Articaine	14 (13.3)
**(n = 105)**	3% Mepivacaine	1 (1)
	3% Prilocaine	1 (1)
**Mode of**	Infiltration	87 (67.4)
**anesthesia**	Inferior alveolar nerve block	32 (24.8)
application *	Intrapulpal	8 (6.2)
**(n = 129)**	Intraligamentary	2 (1.6)
**Amount of**	Less than 1 cartridge	12 (11.4)
anesthesia **	1 cartridge	64 (61.0)
**(n = 105)**	1.5 cartridge	1 (1)
	2 cartridges	22 (20.9)
	More than 2 cartridges	6 (5.7)
**Supplementary**	2% Lidocaine	11 (57.9)
**anesthesia**	4% Articaine	7 (36.8)
**(n = 19)**	3% Mepivacaine	1 (5.3)

**Table 5:** Results from univariable analyses for intraoperative pain (IOP) during treatment visits with categorical variables, frequency, unadjusted odds ratio, and 95% confidence interval (CI) (** *p* < 0.01 and * *p* < 0.05).

Categories	Variables	IOP		Odds Ratio	*p*-Value
		Yes	No	(95% CI)	
**Treatment**	Dental students	18	37	7.62 (2.1–27.9)	<0.01 **
**providers**	Postgraduate candidates and specialists	3	47		
**Symptoms**	Preoperative symptoms	10	20	2.91 (1.1–7.9)	<0.05 *
	No preoperative symptoms	11	64		
**Patients’**	Male	8	36	0.82 (0.3–2.2)	0.69
**gender**	Female	13	48		
**Dental**	Maxilla	11	49	0.79 (0.3–2.1)	0.62
**arch**	Mandible	10	35		
**Tooth**	Anterior	2	9	0.88 (0.2–4.4)	0.87
**type**	Posterior	19	75		
**Type of**	Infiltration only	13	60	0.65 (0.2–1.8)	0.39
**injection**	Inferior alveolar nerve block	8	24		

## Data Availability

The data presented in this study are available on request from the corresponding author due to patient sensitive information.

## Supplementary Materials

The following supporting information can be downloaded at: https://www.mdpi.com/article/10.3390/dj14100647/s1, Supplementary File: The questionnaire.

## References

[1] Närhi M., Bjørndal L., Pigg M., Fristad I., Rethnam Haug S. (2016). Acute dental pain I: Pulpal and dentinal pain. Den Nor. Tann. Tid..

[2] Taylor P.E., Byers M.R., Redd P.E. (1988). Sprouting of CGRP nerve fibers in response to dentin injury in rat molars. Brain Res..

[3] Byers M.R., Taylor P.E., Khayat B.G., Kimberly C.L. (1990). Effects of injury and inflammation on pulpal and periapical nerves. J. Endod..

[4] Rodd H.D., Boissonade F.M. (2000). Substance P expression in human tooth pulp in relation to caries and pain experience. Eur. J. Oral Sci..

[5] Hargreaves K.M., Keiser K. (2002). Local anesthetic failure in endodontics: Mechanisms and Management. Endod. Top..

[6] Nusstein J., Reader A., Nist R., Beck M., Meyers W.J. (1998). Anesthetic efficacy of the supplemental intraosseous injection of 2% lidocaine with 1:100,000 epinephrine in irreversible pulpitis. J. Endod..

[7] Nusstein J.M., Reader A., Drum M. (2010). Local Anesthesia Strategies for the Patient With a “Hot” Tooth. Dent. Clin. N. Am..

[8] Segura-Egea J.J., Cisneros-Cabello R., Llamas-Carreras J.M., Velasco-Ortega E. (2009). Pain associated with root canal treatment. Int. Endod. J..

[9] Demilie A.E., Denu Z.A., Bizuneh Y.B., Gebremedhn E.G. (2024). Incidence and factors associated with failed spinal anaesthesia among patients undergoing surgery: A multi- center prospective observational study. BMC Anesthesiol..

[10] Dagher F.B., Yared G.M., Machtou P. (1997). An evaluation of 2% lidocaine with different concentrations of epinephrine for inferior alveolar nerve block. J. Endod..

[11] American Association of Endodontists Root Canal Explained. https://www.aae.org/patients/root-canal-treatment/what-is-a-root-canal/root-canal-explained/.

[12] Khayat B.G., Byers M.R., Taylor P.E., Mecifi K., Kimberly C.L. (1988). Responses of nerve fibers to pulpal inflammation and periapical lesions in rat molars demonstrated by calcitonin gene-related peptide immunocytochemistry. J. Endod..

[13] Kfir A., Blau-Venezia N., Tsesis I., Goldberger T., Metzger Z. (2017). Does root canal treatment in necrotic pulp or retreatment cases with periapical lesions require anaesthesia? An in vivo clinical study. Int. Endod. J..

[14] Haug S.R., Røegh M., Fristad I. (2024). Endodontic emergency patients’ profile and treatment outcome—A prospective cohort study. BMC Oral Health.

[15] Watkins C.A., Logan H.L., Kirchner H.L. (2002). Anticipated and experienced pain associated with endodontic therapy. J. Am. Dent. Assoc..

[16] Carter A.E., Carter G., Boschen M., AlShwaimi E., George R. (2015). Ethnicity and Pathways of Fear in Endodontics. J. Endod..

[17] Afshari E., Sabbagh S., Khorakian F., Sarraf Shirazi A., Akbarzadeh Baghban A. (2023). Reducing pain and discomfort associated with rubber dam clamp placement in children and adolescents: A systematic review and meta-analysis of effectiveness. BMC Oral Health.

[18] Elmatary A., Moawad E., Heidarifar O., Stone S. (2025). Endodontic access cavity preparation: Challenges and recent advancements. Br. Dent. J..

[19] Byers M.R., Närhi M.V. (1999). Dental injury models: Experimental tools for understanding neuroinflammatory interactions and polymodal nociceptor functions. Crit. Rev. Oral Biol. Med..

[20] Swanljung O., Vehkalahti M.M. (2018). Root Canal Irrigants and Medicaments in Endodontic Malpractice Cases: A Nationwide Longitudinal Observation. J. Endod..

[21] Aggarwal V., Singla M., Subbiya A., Vivekanandhan P., Sharma V., Sharma R., Prakash V., Geethapriya N. (2015). Effect of Preoperative Pain on Inferior Alveolar Nerve Block. Anesth. Prog..

[22] Shroff M., Kishan K.V., Shah N., Volety S., Kaur S., Kotecha N. (2025). Evaluation of three different drugs administered through intraligamentary route for reduction of intraoperative pain of symptomatic irreversible pulpitis in mandibular molars—A randomized triple-blind single-center clinical study. J. Conserv. Dent. Endod..

[23] Khan A.A., Owatz C.B., Schindler W.G., Schwartz S.A., Keiser K., Hargreaves K.M. (2007). Measurement of mechanical allodynia and local anesthetic efficacy in patients with irreversible pulpitis and acute periradicular periodontitis. J. Endod..

[24] Hashemian A.M., Omraninava A., Kakhki A.D., Sharifi M.D., Ahmadi K., Masoumi B., Mehrpour O. (2014). Effectiveness of local anesthesia with lidocaine in chronic opium abusers. J. Emergencies Trauma Shock.

[25] Marti F., Lindner G., Ravioli S. (2022). Resistance to local anaesthetics: A literature review. Br. J. Anaesth..

[26] Becker D.E., Reed K.L. (2012). Local Anesthetics: Review of Pharmacological Considerations. Anesth. Prog..

[27] Malamed S.F., Gagnon S., Leblanc D. (2001). Articaine hydrochloride: A study of the safety of a new amide local anesthetic. J. Am. Dent. Assoc..

[28] Pogrel M.A. (2007). Permanent Nerve Damage from Inferior Alveolar Nerve Blocks—An Update to Include Articaine. J. Calif. Dent. Assoc..

[29] Pan J., Wang Y., Qian Y., Zou J., Zhang Q. (2023). Comparison of dental anesthetic efficacy between the periodontal intraligamentary anesthesia and other infiltration anesthesia: A systematic review and meta-analysis. PeerJ.

[30] Afkhami F., Rostami G., Peters O.A., Kamalian F. (2023). Pulpal anesthesia of maxillary first molars using 4% articaine infiltration in patients with symptomatic irreversible pulpitis: A randomized controlled clinical trial. Clin. Oral Investig..

[31] Hargreaves K.M., Keiser K. (2002). Development of new pain management strategies. J. Dent. Educ..

[32] Suresh N., Arulalan M., Raj G.R., Natanasabapathy V., Abraham S.B., Veettil S.K., Lima T.C.d.S., Bastos J.V., Pulikkotil S.J., Nagendrababu V. (2026). Effect of Oral Corticosteroid Premedication on the Success of Anaesthetising Mandibular Teeth with Irreversible Pulpitis: A Systematic Review with Meta-Analysis and Trial Sequential Analysis of Randomized Clinical Trials. Int. Endod. J..

[33] Khademi A., Iranmanesh P., Mosayebi N., Heydari M., Bagherieh S. (2023). Effect of premedication on the success of inferior alveolar nerve block in patients diagnosed with irreversible pulpitis: An umbrella review. Dent. Res. J..

