# Supplementary material for: Local Anesthetic Use and Intraoperative Pain During Root Canal Treatment: A Cross-Sectional Survey Among Dental Students, Postgraduates, and Endodontists
Source: Dent J (Basel). 2026 Oct 7;14(10):647. doi: 10.3390/dj14100647 (PMC13702915; doi:10.3390/dj14100647)
Supplement: Supplementary file 1 [file dentistry-14-00647-s001.zip › dentistry-4564278-supplementary.pdf]

Supplementary 1, THE QUESTIONNAIRE

Part 1:

This survey aims to investigate the use of local anesthesia during endodontic treatment, including anesthetic techniques, the occurrence and management of insufficient anesthesia and intraoperative pain, and practitioners' perspectives and experiences. The findings may contribute to a better understanding of current clinical practices and help identify areas for future research and education. By participating in the survey, you consent to the use of these responses for research purposes. This study was reviewed by the Regional Committee for Medical and Health Research Ethics (REK 616463).

Please scan the QR code to proceed with the survey. We appreciate your time and valuable participation in this survey.

Part 2:

1. Treatment provider:
   - 4th-year dental student
   - 5th-year dental student
   - Postgraduate student
   - Specialist in Endodontics

2. Patient's gender:
   - Female
   - Male
   - Others: ______

3. Tooth number
   Free text ______

4. Does the patient have symptoms today?
   - Yes
   - No
   - Comments: _____

5. Initial diagnosis of the tooth: Pulp
   - K04.0 Pulpitis
   - K04.1 Pulp necrosis
   - Previously root-filled tooth
   - Others: _____

6. Initial diagnosis of the tooth: Periapical
   - K04.4 Acute apical periodontitis

- K04.5 Chronic apical periodontitis
- K04.6 Abscess with sinus tract
- K04.7 Abscess without sinus tract
- No periapical lesion
- Others:_____

7. Which treatment session is it today: Visit #
- 1
- 2
- 3
- More than 3

8. What type of treatment is being performed today?
- Emergency treatment
- Access cavity preparation
- Instrumentation
- Obturation
- Others: _____

9. Did you use anesthesia during the treatment?
- Yes
- No

Part 3:

10. Type of local anesthesia:
- Xylocaine dental adrenalin
- Septocaine
- Carbocaine dental
- Others: _____

11. Amount of anesthesia:
- Less than 1 carpule
- 1 carpule
- 2 carpuler
- More
- Others: _____

12. How was the anesthesia administered:
- Infiltration
- Inferior alveolar nerve block
- Intraligamentary
- Intrapulpal
- Others: _____

13. Was the anesthesia sufficient
- Yes
- No

14. Was there a need for additional or supplementary local anesthesia during the treatment?
- Yes
- No

Part 4:

15. Did patients experience any pain during the treatment?
    - Yes
    - No

16. Do you think anesthesia was necessary during the treatment?
    - Yes
    - No
    - Perhaps yes
    - Perhaps no
    - Others: _____

17. Comments
    - “Free text”: _____
